# Overall impact of outpatient stress cardiac magnetic resonance (CMR) imaging on clinical care is independent of appropriate use criteria

**DOI:** 10.1186/1532-429X-18-S1-Q20

**Published:** 2016-01-27

**Authors:** Sloane A McGraw, Omer Mirza, Vibhav Rangarajan, Satish J Chacko, Michael A Bauml, Afshin Farzaneh-Far

**Affiliations:** 1 Cardiology, University of Illinois- Chicago, Chicago, IL USA; 2 Cardiology, Duke University, Durham, NC USA

## Background

Stress cardiac-magnetic-resonance (CMR) imaging can provide important diagnostic and prognostic information in patients with known or suspected coronary artery disease. However, given the rising costs of imaging, there is increasing pressure to provide evidence for direct additive impact on clinical care. Appropriate use criteria (AUC) have been developed by professional organizations as a response to rising costs, with the goal of optimizing test-patient selection. Consequently, the AUC are now increasingly used by third-party-payers to assess reimbursement. However, these criteria were created by expert consensus with limited systematic validation. The aim of this study was to determine whether the AUC can predict rates of active change in clinical care resulting from stress CMR.

## Methods

305 consecutive outpatients referred from cardiology clinic for stress CMR were prospectively enrolled at a U.S. university teaching hospital. Definitions for "active change in clinical care" due to stress CMR were pre-defined and classified by two cardiologists directly from medical records and/or from patients. Categories included: coronary angiography, revascularization, pre-operative clearance, medication change, subspecialty referral, ordering of additional diagnostic testing, and discharge from cardiology clinic. Tests were classified as "appropriate", "may be appropriate" or "rarely appropriate" according to the "2013 Multimodality Appropriate Use Criteria for the Detection and Risk Assessment of Stable Ischemic Heart Disease."

## Results

**Figure 1:**
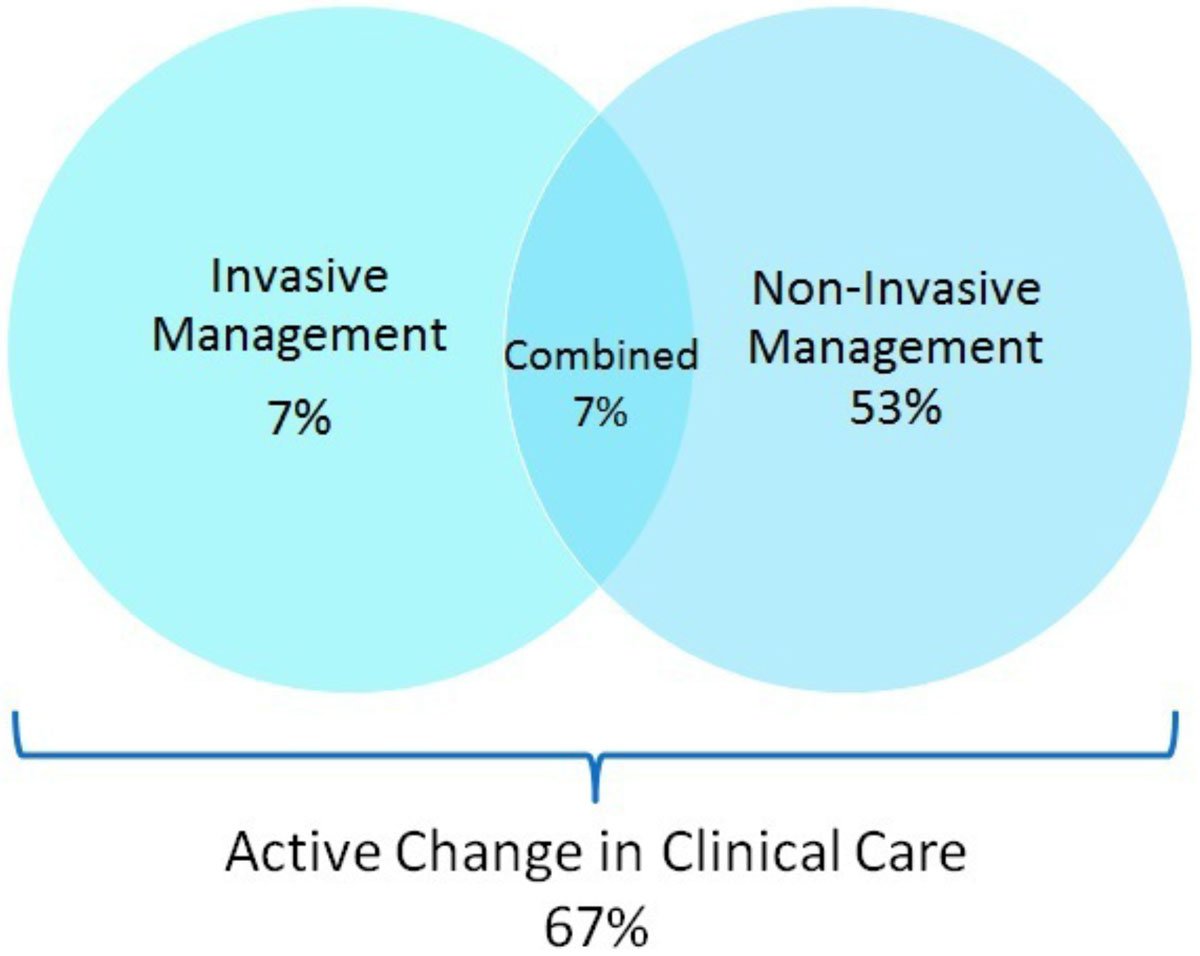
**Overall Active Change in Clinical Care Due to Stress CMR**

**Figure 2:**
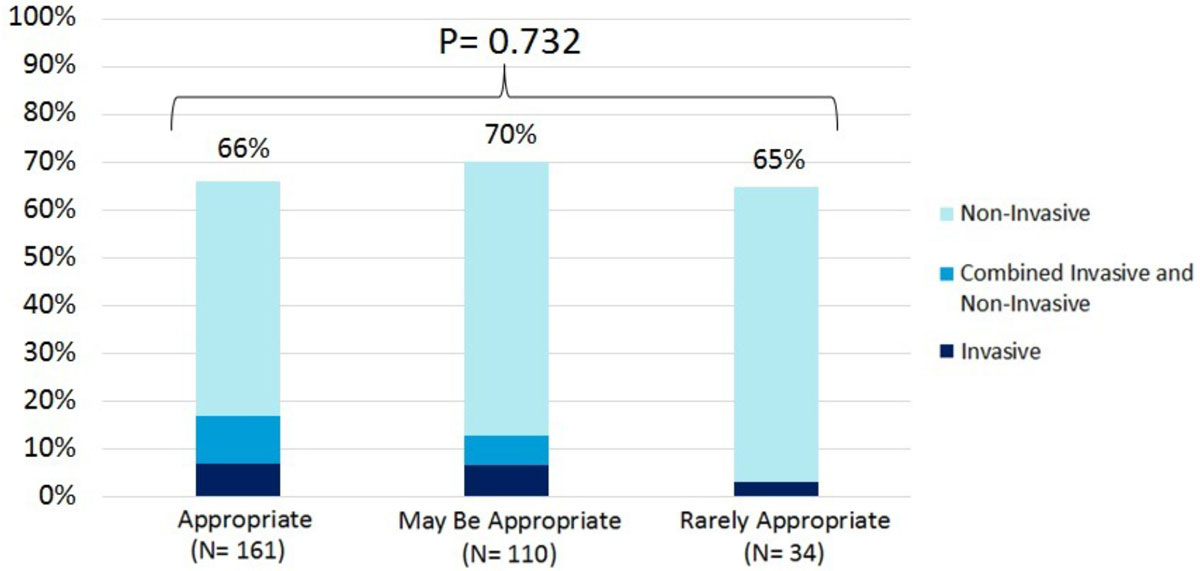
**Active Change in Clinical Care Based on AUC Classification**

Overall, stress CMR led to an active change in clinical care in 67% of patients. This included initiation of invasive management in 14% of cases, and non-invasive changes in 60% (Figure 1). Stress CMR results directly led to coronary angiography in 14%, revascularization in 5%, pre-operative clearance in 12%, medication change in 18%, subspecialty referral in 9%, ordering of additional diagnostic testing in 9%, and discharge from cardiology clinic in 18%. Rates of active change in clinical care did not vary significantly across AUC categories (p = 0.732) (Figure 2).

## Conclusions

Stress CMR made a significant clinical impact on management, resulting in active change in clinical care in 67% of patients, with the majority of changes being non-invasive. However, overall rates of active change in clinical care were similar across AUC categories (p = 0.732). This suggests that consideration should be given to upgrading some of the "rarely appropriate" indications to either "may be appropriate" or "appropriate" classifications.

